# Fighting Like Cats and Dogs: Challenges in Domestic Carnivore Oocyte Development and Promises of Innovative Culture Systems

**DOI:** 10.3390/ani11072135

**Published:** 2021-07-19

**Authors:** Martina Colombo, Isa Mohammed Alkali, Sylwia Prochowska, Gaia Cecilia Luvoni

**Affiliations:** 1 Dipartimento di Scienze Veterinarie per la Salute, la Produzione Animale e la Sicurezza Alimentare “Carlo Cantoni”, Università degli Studi di Milano, 26900 Lodi, Italy; isa.mohammed@unimi.it (I.M.A.); cecilia.luvoni@unimi.it (G.C.L.); 2 Department of Reproduction and Clinic of Farm Animals, Wrocław University of Environmental and Life Sciences, Grunwaldzki Square 49, 50-366 Wrocław, Poland; sylwia.prochowska@upwr.edu.pl

**Keywords:** canine, feline, co-culture, IVC, IVF, IVM, microfluidic, 3D

## Abstract

**Simple Summary:**

In assisted reproduction, in vitro embryo production is the generation of embryos from isolated gametes in an equipped laboratory. This procedure is commonly employed in humans as well as in animals to preserve or improve their fertility, but while it works well in some species, such as cattle, it still faces some challenges in others, such as cats and dogs, which are important animal models to develop assisted reproduction techniques for related wild, endangered species. Traditionally, gametes and embryos are cultured in vitro on glass or plastic dishes, but these supports are very different from the ovarian follicles, the oviducts, or the uterus, which are the physiological environments where oocytes and embryos grow and develop. This review article describes these culture systems, the cellular alterations that could arise from their use, and it illustrates innovative possibilities (e.g., three-dimensional or microfluidic cultures) that could improve the outcomes of in vitro cultured feline and canine reproductive cells.

**Abstract:**

In vitro embryo production in cats and dogs still presents some challenges, and it needs to be optimized to transfer efficient protocols to related wild, endangered species. While the chemical composition of culture media has been the focus of several studies, the importance of culture substrates for oocyte and embryo culture has often been neglected. Traditional in vitro systems, i.e., two-dimensional cultures, do not resemble the physiological environments where cells develop, and they may cause morphological and functional alterations to oocytes and embryos. More modern three-dimensional and microfluidic culture system better mimic the structure and the stimuli found in in vivo conditions, and they could better support the development of oocytes and embryos in vitro, as well as the maintenance of more physiological behaviors. This review describes the different culture systems tested for domestic carnivore reproductive cells along the years, and it summarizes their effects on cultured cells with the purpose of analyzing innovative options to improve in vitro embryo production outcomes.

## 1. Introduction

Due to the increasing number of animal species threatened with extinction [1], the use of animal models to study reproduction physiology and develop new assisted reproductive technologies (ARTs) is gaining more and more importance. Domestic cats (*Felis catus*) and dogs (*Canis lupus familiaris*) are the models of choice for wild felids and canids, and some of the protocols designed in the tame species have already been successfully transferred to their wild counterparts [2,3].

However, ARTs in carnivores are not as efficient as in other species. In vitro embryo production (IVEP) has been applied in cattle, horses, pigs, mice and humans for many years, and it allowed the achievement of significantly improved protocols. Especially in the bovine, IVEP is a widespread, efficient method applied both for research and commercial purposes, which gives outstanding results [4]. Instead, in domestic carnivores, many challenges in the specific reproductive physiology, especially in the dog, limited the development of these techniques, which are not routinely applied also due to their costs [5,6]. Therefore, new insights to enhance oocyte in vitro outcomes are still needed. Some hope could be given by innovative and three-dimensional (3D) culture systems, which were developed in the last few decades to supply cultured cells with more physiological living conditions and were also applied to gametes and embryos.

The aim of this review is to illustrate the past and current in vitro culture methods for domestic cat and dog oocytes and embryos, the most recent advancements in this field and the possibilities that the near future could offer to improve in vitro embryo production systems in these species.

## 2. In Vitro Embryo Production in Cats and Dogs

The most common source of female gametes from queens and bitches is immature oocytes from isolated ovaries obtained after routine spaying, and these will be the main subject of this review. Such collected gametes have to undergo in vitro maturation (IVM) as the first step in IVEP. This is followed by in vitro fertilization (IVF) and embryo in vitro culture (IVC), that would hopefully lead to the formation of embryos which can be transferred to recipient animals [4] or cryopreserved for future use.

Among IVEP steps, IVM is still the most critical phase in domestic carnivores, since metaphase II (MII) rates limit the number of embryos that can be obtained. In cats, MII rates get to around 60% [7], way lower than, for instance, in the bovine, where they usually reach 90% [8]. In dogs, maturation rates barely reach 30% [9], and this might be due to the peculiar reproductive physiology of the bitch, that current IVM systems still fail to resemble. Indeed, dog oocytes are ovulated at an immature stage and need 2–3 days in the oviduct, with a high progesterone concentration, to mature [5,10].

Following maturation, oocytes can be fertilized. Epididymal or urethral spermatozoa, more commonly in the cat, or ejaculated semen, more commonly in the dog, can be used for IVF. Although lower than in other species (e.g., bovine and mice [11,12]), efficiency is good in cats, where fertilization rates go beyond 50% of collected immature oocytes [13], while IVF is still tentative in dogs. Fertilization failure and polyspermy, which may also be linked to IVM challenges (e.g., poor IVM rates during the prolonged anestrus period [14], scarce cytoplasmic maturation), limit dog IVF efficiency, and less than 10% of collected oocytes undergo proper fertilization, defined as the formation of two pronuclei [9].

Cleavage and further embryo development proceed in the domestic cat, and around half of the cleaved embryos become a morula or a blastocyst [7,13]. Instead, development is usually blocked at the four to eight cell stage in dogs [15], where the formation of blastocysts is uncommon and sporadic, and so far it was only accomplished when embryo IVC was performed in association with somatic cells [16,17]. As a consequence, it is easy to imagine that live kittens from in vitro matured and fertilized oocytes have been obtained more than once [18,19], whereas in dogs births were obtained only after IVF of in vivo-matured oocytes [20], and the scientific community is still waiting for IVM-IVF puppies.

Unfortunately, little progress has been made along the years with traditional culture systems, which are based on specific media deposited as drops in Petri dishes or multi-well plates. To improve in vitro outcomes, several culture media were experimented for IVM and IVC (e.g., sequential or with different supplementations) both in cats [21,22,23,24,25] and in dogs [26,27,28], but so far this did not appear to be enough to support the developmental competence of domestic carnivore oocytes.

## 3. Traditional Culture Systems

### 3.1. Culture Media

For a long time, the culture medium alone has been considered the culture environment. Most of the research efforts to improve the in vitro culture conditions for oocytes and embryos have traditionally been focused on the variation of the chemical composition of the culture media or on the addition of specific molecules with putative beneficial effects.

Once understood that mimicking the physiological environment was the key to promote oocyte maturation in vitro [7], several supplementations were tested. For cat oocytes, attempts were made to design culture media which could resemble the follicular fluid. Addition of hormones (follicle-stimulating hormone—FSH, and luteinizing hormone—LH)) of different origin at different concentrations was tried to find the suitable combinations [29,30]. Porcine or human hormones, at 0.02–0.5 IU/mL, are usually employed. The study of different protein supplementations led to the consensus that bovine serum albumin (BSA) is suitable, whereas fetal bovine serum (FBS) or fetal calf serum (FCS) could inhibit oocyte maturation [31,32,33,34]. Supplementation of growth factors such as epidermal growth factor (EGF) or insulin-like growth factor-I (IGF-I) contributes to maturation [23] and embryo development [21,22]. Different antioxidants, including cysteine, have also been tested to mitigate the oxidative stress derived from the in vitro conditions and to regulate glutathione (GSH) balance [29,35,36]. Although they are not always included as a standard supplementation in IVM media, they could improve oocyte developmental competence [29,36].

For dog oocytes, as mentioned, maturation takes longer and physiologically occurs in the oviduct. To mimic these conditions, extended IVM lengths and sequential media were tested, but the optimal combination has not been found yet. Whereas IVM lasts 48 or more commonly 72 h (for a review see [37]), prolonged IVM (up to 96 h [26,38]) was also experimented, even if its benefits on maturation outcomes remained controversial. Based on the few studies that investigated embryo development after fertilization, the best IVM length seemed to be 48 h [39]. As in the cat, several chemical supplements were evaluated, following similar rationales and considering that the medium composition could be adapted during the culture to the different needs of maturing oocytes. Besides FSH and LH, that did not promote meiotic maturation of dog oocytes when present for the whole length of the culture [40], similarly to other gonadotropins (i.e., equine chorionic gonadotropin (eCG) or human chorionic gonadotropin (hCG) [41]), other compounds were experimented to better mimic the peculiar hormonal conditions in which dog oocytes mature (i.e., decreasing estradiol-17β and increasing progesterone concentration [39,42]). Estrogen and progesterone were then used, but conflicting results were obtained, also due to the estrous phase of the bitch from which the ovaries were collected [42,43,44]. The dynamic endocrine environment where dog oocytes mature prompted the creation of multi-step culture systems where hormonal supplementation could change during culture. For instance, the addition of hCG only in the first half of 96 h IVM improved maturation rates [26], while the use of hCG followed by progesterone in another bi-phasic system promoted cytoplasmic maturation in MII oocytes [28]. Likewise, the study of protein supplementations is also controversial. Bovine serum albumin and different kinds of sera (e.g., FBS, bitch serum collected from dogs at different estrous cycle stages, estrous cow serum) were tested [45,46,47], but some studies reported that protein supplementation is not essential for dog oocyte IVM [48,49,50]. Finally, the use of growth factors such as EGF [51,52,53], IGF-I [54], or growth differentiation factor 9 (GDF-9) and bone morphogenetic protein 15 (BMP-15) [55] was attempted and it benefitted meiosis resumption [52] and full maturation rates [51,53,54,55]. Similarly, the addition of antioxidants, including thiols [51,56], retinoic acid [57], and more recently L-carnitine [58], was proved to improve IVM outcomes [51,57,58], since it is believed that dog oocytes are very sensitive to oxidative stress due to the huge amount of intracellular lipids [39].

For embryos, once again, culture media were designed to resemble the environment where early embryonic development occurs. For cats, one-step [29,59,60] or multi-step [18,19,24,25,61,62,63,64] IVC systems have been employed. While the former have the advantage of reducing embryo manipulation, while the latter offer the possibility to adapt the nutrient supply according to the developmental stage of the embryos. While early embryos seem to benefit from the presence of BSA and non-essential amino acids (NEAA), at more advanced stages of development essential amino acids (EAA) and FBS are generally added [13,24] to stimulate development and increase cell number. Attempts to better characterize the specific needs of feline embryos were also made, both investigating the distribution of proteins in the cat oviduct [65] and the embryo development and metabolism following culture in different media [24]. As a result, a feline-optimized culture medium (FOCM) was designed to contain, among the other compounds, alanyl-glutamine and taurine [24]. Compared to glutamine, the use of alanyl-glutamine reduces the production of potentially toxic NH_4_ [66], whereas taurine could act as an osmolyte and mitigate the inhibitory effects of NaCl, which has to be present at low concentrations for feline embryo development [24,67].

It is also worth mentioning that, both for IVM and IVC in the domestic cat, commercial media were experimented to increase reproducibility and repeatability and reduce the workload in the laboratory, and they were suitable for IVEP as well as lab-made media [68]. In this species, differences in the culture atmosphere during IVC were also investigated. Although 5% CO_2_ in air is the most common condition, lower concentrations of oxygen (5%) promote the in vitro development of cat embryos [69].

Despite the difficulties to obtain in vitro-derived embryos in the dog, some shared features can be found in the IVC media of the few studies on dog IVEP. Media are usually similar to those used for IVM, and they employ a base medium with serum as a protein source [16,17].

### 3.2. Culture Substrates

Despite several studies and the fact that, at least for cat oocytes and embryos, IVM and IVC media currently employed in different labs are quite similar, developmental rates of carnivores’ gametes are still somehow unsatisfactory. One of the reasons could be that the physical support where cells grow was not regarded to the same extent as culture media, even though it can influence the culture microenvironment as much as the chemical compounds [7]. Two-dimensional (2D) in vitro culture systems have been traditionally employed for oocyte and embryo culture due to their efficiency, convenience, affordability, and ease of use [70,71,72]. The supports are usually disposable and made of plastic, generally polystyrene, while glass was more common in the past. For IVEP, they include Petri dishes and multi-well plates, where cells grow on the flat bottom, completely immersed in culture medium [73]. Cell growth substrates can be characterized according to some physical properties, such as roughness, elasticity and topography [74]. Polystyrene is usually chosen for the production of cell culture dishes thanks to its optical clarity, easy manufacturing and reasonable cost [73], but it is much stiffer than the surfaces the cells stay in contact with in their in vivo environment [75] and it also has an influence on cell growth.

In vivo, cells are surrounded by the extracellular matrix (ECM), which is a complex milieu composed by structural proteins, proteoglycans, glycoproteins and other molecules. The ECM enables the spatial organization of cells and tissues, regulating many essential cellular behaviors, including adhesion, migration, proliferation and differentiation [76] thanks to its mechanical properties. These depend on the localization and composition of the ECM, which is mainly made of proteins, such as collagen, elastin and laminin, that can create a network between the enclosed cells [77]. In the ECM, cells are also connected to each other through specific surface receptors, such as integrins, forming 3D structures or tissues [77] in a microenvironment where gradients of deformability of surrounding material are present [74]. Extracellular matrix stiffness greatly varies among different tissues or cellular regions [78] and can also direct cell fate [79]. Recreating these conditions in the lab is still challenging.

In vitro, cells can perceive the differences in substrate geometry, roughness and stiffness supplied by standard culture dishes [80], which do not resemble the ECM. Cell culture supports also lack gradients of signaling molecules, oxygen, nutrients and catabolites [81]. As a consequence, cell physiology and morphology change, and cells modify their shape, subcellular organelles or their behavior, including adhesion to the substrate, migration and differentiation [82]. In an attempt to adapt to the 2D environment, cells flatten on the surface of the dish, because of the remodeling of their cytoskeleton [83], also losing their polarity. Modifications in the nuclear shape and alterations in gene expression and protein synthesis can also occur in 2D-cultured cells compared to in vivo-living cells [84,85]. Furthermore, cell could also lose membrane receptors and undergo changes in their response to hormones, stimuli and secretions [71].

Finally, it should be considered that in vitro culture systems are static conditions, in which it is challenging to recreate a proper air-liquid interface [71] and physical forces acting on the cells. While in vivo the ECM and the body fluids cause mechanical and shear stress on the cells, which are converted in intracellular biochemical signals influencing cell behavior [71], the presence of still culture medium does not supply these signals in vitro. In addition, fluid movement refreshes the surrounding microenvironment, bringing and enhancing the distribution and availability of new nutrients and removing potentially toxic metabolites [71]. Recreating this setting in vitro should be a priority to guarantee proper cellular growth and development.

Two-dimensional culture systems cause peculiar alterations in oocyte/embryo morphology and physiology also due to the fact that they are usually put in culture as “Multicellular systems”. Indeed, oocytes during IVM are usually cultured as cumulus-oocyte complexes (COCs), where the gamete is surrounded by somatic cells, while embryos are multicellular by definition, since along IVC the number of blastomeres increases. In both cases, communication among different cells is vital to obtain satisfactory IVEP outcomes. Although the actual influence of 2D culture conditions on cat and dog reproductive cells remains to be investigated, in other species some observations were done. In COCs, the 2D arrangement of traditional culture systems, such as medium microdrops, might disrupt the cellular communications between the oocyte and its cumulus cells, might modify the polarity and secretion of both germinal and somatic cells, might lead to a distortion of the cell-to-cell orientation, and might bring about an abnormal distribution of paracrine factors [86,87,88,89]. In embryos, 2D culture systems might cause morphological alterations, might not support morphological changes typical of embryo development, might damage the cell-to-cell communications between blastomeres and the embryo 3D architecture, and might lead to an abnormal gene expression compared to in vivo-derived embryos [90,91].

## 4. Alternative Culture Systems

### 4.1. Companion Cells and Cell-Derived Products for the Enrichment of Culture Conditions

The search for better culture conditions, which could mimic more faithfully the physiological environment where the cells grow and allow them to preserve an in vivo-like functionality, is still in progress. Historically, there were some attempts to enrich the in vitro 2D conditions with the use of co-cultures. Co-cultures were used to recreate the physiological intercellular communications, since in vivo cells interact with each other in complex systems. The co-existence of different cell types stimulates signaling and cross-talking through soluble factors or direct cell-to-cell contacts [92] and can be considered a physico-chemical enrichment to the culture environment. Indeed, co-cultured cells can interact through paracrine signals, and the sharing of soluble factors, such as growth factors, might ameliorate the culture environment and improve cell development [93,94]. There might also be cell-contact dependent effects [92] and companion cells could offer a physical support, as in the case of feeder cell monolayers.

Probably, the most known use of co-cultures in IVEP is the addition of companion cells for embryo culture. Although some trials gave no effects or were even detrimental, most of the times this approach could improve embryo yield and quality, as well as pregnancy rates, thanks to the several beneficial effects that co-cultured cells can supply, including secretion of embryotrophic molecules, modulation of nutrient profile, removal of toxic substances and protection versus oxidation and other in vitro culture-derived stressors [95]. Several types of cells have been employed for this purpose, including tubal cells, granulosa or cumulus cells, fibroblasts and different epithelial cells, but it seemed that the best results were obtained with oviductal cells co-cultures, probably because of their involvement in the physiological early embryo development [95]. Moreover, in humans, co-cultures also appeared beneficial for the rescue of poor-quality embryos [96] or those that underwent stressing procedures, such as cryopreservation or micromanipulation [97,98].

Embryo co-cultures have also been applied to domestic carnivores (Table 1). In cats, oviductal cell co-culture was not beneficial for embryo development [99], while it gave better outcomes if employed only after 72 h, even though it was not enough to promote the development into blastocysts of cat morulae [100]. In dogs, IVC with murine embryonic fibroblasts allowed embryo development until the morula stage, which is an outstanding result for in vitro matured canine oocytes [17]. Similarly, co-culture with bovine cumulus cells allowed development of a blastocyst in vitro [16]. In cats, co-culture with good quality homospecific or heterospecific (i.e., murine) companion embryos was also attempted, and it improved embryo development and quality [101,102].

With the same rationale, co-cultures have also been used for IVM (Table 1). Among signaling molecules that can be exchanged by co-cultured cells, oocyte-secreted factors (OSFs) are compounds produced and detected by COCs, that in response can modulate their own metabolism and that of the surrounding cumulus [94,103]. In mammals, some OSFs stimulate oocyte competence [94,104,105], whereas others, such as the well-known GDF-9 and BMP-15, exert their action on cumulus cells and regulate their function, proliferation, differentiation and gene expression [94,103,106,107]. Therefore, co-culture of immature oocytes, especially low competence oocytes, with other COCs can be a strategy to improve maturation rates. In the domestic cat, the co-culture of denuded oocytes with intact COCs gave various results. While it did not seem beneficial for full maturation [108], its influence on embryonic developmental rates is controversial [109,110].

Enrichment of the IVM microenvironment with companion somatic cells has also been applied. Oviductal cells in monolayers were especially used in the dog, to recreate the peculiar environment where the oocytes of this species mature [111,112,113,114,115], and they were generally beneficial for maturation rates, sometimes with increases of about 10% on MII rates [112,115]. Similarly, in vitro maturation of dog oocytes in isolated oviducts improved meiosis resumption [116]. Granulosa cells were also used, both for dog [117] and cat oocytes [108,110]. In dogs, bovine granulosa cell monolayers could improve MII rates by 5–20% of COCs and denuded oocytes, while canine granulosa cell monolayers gave poorer results [117]. In cats, granulosa cells were somehow beneficial to partial meiosis resumption, but not full maturation of denuded oocytes [108,110] nor their embryo development [110]. Other cell types were also used, and co-culture with embryonic fibroblasts of canine or murine origin improved cytoplasmic and nuclear maturation of canine oocytes with an increase of 6–8% in MII rates [17].

**Table 1:** Two-dimensional co-culture systems tested for cat and dog oocytes and embryos.

Species	Cellular Target	Co-Culture System/Companion Cells	Outcome	Reference
Domestic dog	COCs	Canine oviductal cells	Improved in vitro maturation	[111,112,113,114,115]
	COCs	Canine isolated oviduct	Better resumption of meiosis	[116]
	COCs and denuded oocytes	Bovine and canine granulosa cell monolayers	Improved maturation rates with bovine cells	[117]
	COCs and embryos	Bovine cumulus cell monolayer	Improved oocyte maturation and embryo development (until the blastocyst stage)	[16]
	COCs and embryos	Murine and canine embryonic fibroblasts	Improved oocyte maturation and embryo development (until the morula stage)	[17]
Domestic cat	Denuded oocytes	Feline COCs	Improved maturation and embryo development	[108,109]
	Denuded oocytes	Feline cumulus cells	No improvement in maturation or embryo development	[110]
	In vivo matured COCs and embryos	Feline oviductal cell monolayer	No improvement in fertilization or embryo development	[99,100]
	Embryos	More advanced (older) feline embryos	Improved embryo development	[101]
	Embryos	Excellent quality feline, mouse or cattle embryos	Improved embryo development	[102]

COCs—cumulus-oocyte complexes.

Finally, other cellular or bodily products have been studied in another attempt to improve IVEP outcomes. For instance, the use of follicular fluid can be beneficial for the IVM outcomes of canine oocytes [118], probably thanks to its content of proteins, hormones, growth factors and cytokines [119] that can regulate oocyte activity. The addition of exogenous OSFs, similarly, could have a positive effect on IVM. In the same species, the simultaneous supplementation of GDF-9 and BMP-15 improved MII rates, while the blockage of the same molecules with specific antibodies was detrimental for meiosis resumption [55]. Recently, a huge recognition was also given to extracellular vesicles and their putative beneficial effects (for a review, see [120]). Indeed, these vesicles originating from the plasma membrane, contain several molecules, such as proteins, lipids, and genetic materials, and can stimulate cellular functions and take part into intercellular communication. Extracellular vesicles have also been detected in follicular fluid, thus they might be involved in oocyte maturation and, for this reason, their supplementation during IVM was tested in several species. In dogs, oviductal extracellular vesicles exerted positive effects on the maturation of fresh oocytes, with increases up to 13% in MII rates [121,122]. Instead, in cats, extracellular vesicles have only been tested on immature cryopreserved oocytes. Extracellular vesicles isolated from cat follicular fluid were characterized and supplemented to vitrification/warming media. Immature COCs were able to internalize these vesicles and likely to exploit their content (e.g., proteins, lipids, DNA fragments, RNAs and microRNAs), and as a result an enhanced meiosis resumption was obtained after oocyte warming [123].

All of these approaches, however, present some limits, especially because they are based on the use of companion cells or their products, that are not defined. While the use of chemically defined culture media would allow the use of animal products to be avoided and the standardization of media composition, the use of co-cultures or cellular products leads to some more variability. Co-cultured cells could cause differences in different replicates, they could bring contamination and they are also likely to hinder the repeatability and reproducibility of the experiments. Finally, companion cells or extracellular vesicles usually have to be prepared in advance to be ready on the day of use. Therefore, the creation of such (co-)culture systems is time-consuming and requires planning to fit well in the laboratory routine.

### 4.2. Recent Advances in In Vitro Culture Technology

More recently, researchers have started to employ 3D culture systems to get closer to the in vivo conditions of cell growth. The aim is to recreate the structural features of the ECM and to overcome the limits of 2D substrates. Modern 3D systems, named scaffolds, can provide a suitable environment for cell survival, growth, differentiation and activities, maintaining a morpho-physiology that strongly resembles the cellular shape and behavior observed in vivo [77,83]. These systems should allow a proper spatial organization of cells as well as the production of secreted factors [89], and usually improve viability, response to stimuli, intercellular communication, cell polarization, gene expression and protein synthesis of cultured cells [81].

Scaffolds can be produced by specific biomaterials, which are intended to be biocompatible and not toxic [124]. They can have variable mechanical features, including elasticity, porosity and viscosity, that can be tuned according to the material and its concentration and that should allow cell growth and proliferation for the specific cultured cell type, as well as gas exchange, diffusion of nutrients and removal of cellular waste [89]. The origin of biomaterials for 3D scaffolds can be natural or synthetic. Natural biomaterials are often derived from ECM components (e.g., collagen, fibrin, hyaluronic acid), but they can also be made of other natural substances, including silk, agarose, gelatin and alginate. Instead, synthetic biomaterials include, for instance, polymers, titanium, ceramic-based materials and self-assembled peptides, which are less cell-compatible since they lack cell adhesion sites, have a lower water content and are less likely to incorporate biologically active compounds, but are more reproducible and have a defined composition [83].

Considering the advantages that they offer to cultured cells, 3D systems have also been employed for oocytes and embryos. The first application was the use of a 3D alginate hydrogel for the culture of granulosa cell–oocyte complexes in the mouse [125]. Growing immature murine oocytes were able to grow and develop the structural features of mature oocytes, including cortical granules and a well-formed zona pellucida, as well as to resume meiosis after culture in the alginate beads, while granulosa cells were free to proliferate [125]. Since then, 3D cultures of reproductive cells gained popularity, and different scaffolds were used, including, but not limited to, alginate and Matrigel [126]. The increase in their use has been due to the fact that 3D systems not only maintain oocyte morpho-physiology, but they also support nuclear and cytoplasmic maturation, allowing gamete development into viable progeny after IVF, IVC and embryo transfer into recipients in mice [87,127]. In addition, 3D cultures better maintain the intercellular communications between blastomeres during embryonic development and they promote an in vivo-like genetic expression, as reported in swine, bovine and murine models [90,91,128]. Among the biomaterials that were tested along the years, alginate often proved its suitability for oocytes and embryos. Alginate is a natural anionic polymer produced from alginic acid, a component of the cellular wall and intercellular spaces of brown algae of the genus *Laminaria*, in which it acts as a sustaining skeleton that provides resistance and flexibility to the algae tissues. In research labs, it is appreciated because it does not interfere with cellular functions, it allows the movement of biomolecules, it is transparent and allows microscopic observations, it is biocompatible, it has a low toxicity and a low cost [129,130,131,132,133]. Its use can especially be appreciated for reproductive cells because of its stiffness, porosity, and lack of cell adhesion sites, which allow the creation of a matrix in which the cells do not suffer extreme mechanical stresses, can exchange nutrients and do not unnaturally adhere to the substrate. Indeed, alginate was also the most used biomaterial for cat and dog oocytes and embryos.

The applications of 3D culture systems for domestic carnivores IVEP, which are summarized in Table 2, include both oocyte and embryo culture. For what concerns the dog, the prolonged duration of the IVM causes worries because oocytes have a long time to flatten and adhere to the culture substrate. So, fresh dog COCs were in vitro matured in barium alginate microcapsules to evaluate the effects of 3D culture, and their viability, nuclear status and expression of one selected OSF, that was GDF-9, were assessed [134]. Although viability and MII rates after 72 h of IVM did not differ, the 3D system maintained higher proportions of intact nuclei and better supported meiosis resumption [134]. Expression of GDF-9 decreased during culture in 3D microcapsules, as expected with meiosis resumption [134], but molecular mechanisms of gene expression in 3D compared to 2D conditions remain to be elucidated.

**Figure 1:**
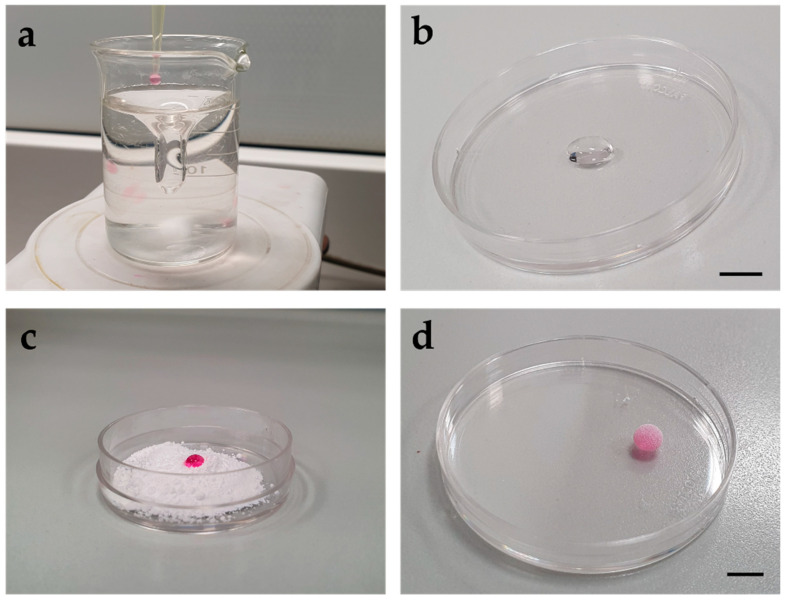
Macroscopic appearance of three-dimensional (3D) culture systems suitable for cat oocyte and embryo culture. (**a**,**b**) Preparation and final appearance, respectively, of 3D barium alginate microcapsules obtained by dropping of 10 µL drops of base culture medium with BaCl_2_ (40 mM) into stirring sodium alginate (0.5%). Black bar: 5 mm. (**c**,**d**) Preparation and final appearance, respectively, of 3D liquid marble microbioreactors obtained by dropping and rolling of 30 µL medium microdrops containing the oocytes onto polytetrafluoroethylene (PTFE) powder. Black bar: 5 mm.

In the cat, taken into account that enriched culture systems could be especially useful for gametes with a lower developmental competence, 3D systems were also tested on denuded and cryopreserved oocytes. Syringe-dropped barium alginate microcapsules were able to support survival and meiosis resumption of fresh COCs as well as the traditional 2D system (i.e., medium microdrops) [135]. In an attempt to further enhance the beneficial effects of 3D culture systems, they were also applied in association with co-culture to combine physical and chemical enrichments to the culture milieu. Co-culture of denuded oocytes with fresh COCs in alginate microcapsules allowed higher viability than the 2D co-culture or the 3D culture without companion cells [135]. The same co-culture was also beneficial for the embryo development of companion COCs, probably due to the exchange of OSFs [136]. However, cat cryopreserved oocytes did not benefit from the 3D system in the same way, probably due to the cryoinjuries which can severely compromise their developmental competence. Standardized 10 µL barium alginate microcapsules (Figure 1a,b), were used for the IVM of cat vitrified oocytes and the IVC of the deriving embryos, resulting in maturation and embryonic developmental rates that were similar to those of the 2D system [137]. Later, the same biomaterial was used for the creation of follicle-like structures, that were microcapsules containing cat granulosa cells and that could better resemble the physiological environment of oocyte maturation [138]. Granulosa cells in 3D culture differed from those in 2D monolayers concerning estradiol and progesterone secretion, which also tended to increase at different extents during culture in the two systems [138]. The presence of granulosa cells, however, did not influence metaphase II outcomes of vitrified oocytes in any culture system [138].

The use of other 3D environments, known as liquid marble microbioreactors, was also tested for cat vitrified oocytes. Liquid marbles (Figure 1c,d) are non-stick liquid droplets, where a liquid core covered by a hydrophobic or hydrophilic powder made of micro- or nano-particles forms a 3D milieu [139]. When they are used for culture applications, such as cancer or stem cell in vitro growth, cells can survive, proliferate, interact and have gaseous exchanges with the outer environment, as well as interact with each other freely and avoid attachment to the bottom of the plate [139,140,141]. This makes liquid marbles an interesting alternative to 2D cultures. Liquid marbles have been already employed in ARTs, for instance for the IVM of sheep oocytes, and they were able to support meiotic resumption and subsequent embryo development as well as the 2D system [142]. Similarly, their use for the IVM of feline oocyte was suitable and as effective as the 2D control for meiosis resumption outcomes [143], and liquid marbles have potential to be applied in other ARTs, such as embryo and follicle IVC or cryopreservation [142,144].

**Table 2:** Existing three-dimensional (3D) culture systems for cat and dog oocytes and embryos.

Species	Cellular Target	Outcome	Reference
Domestic dog	Fresh oocytes	Oocyte maturation in 3D alginate system	[134]
Domestic cat	Denuded oocytes & deriving embryos	Oocyte maturation in 3D alginate system enriched with fresh oocytes & embryo development in 3D	[135,136]
	Vitrified oocytes & deriving embryos	Oocyte maturation in 3D alginate system enriched with fresh oocytes & embryo development in 3D	[137]
	Vitrified oocytes	Oocyte maturation in 3D follicle-like structure (alginate + granulosa cells)	[138]
	Vitrified oocytes	Oocyte maturation in 3D liquid marble microbioreactors	[143]

A current hot topic in alternative culture systems is the use of microfluidics. Known also as organ-on-a-chip or lab-on-a-chip, microfluidic systems can combine 3D architectures, different types of cells and fluid flow, creating a dynamic culture environment [145] that could resemble in vivo conditions even better, especially for the movement of nutrients, metabolites and gases [146]. In ARTs, microfluidic chips could be an impressive tool for several procedures (for two recent reviews on the topic, see [147] and [148]), including IVM, IVF and IVC. Although oocyte maturation and embryo culture of feline and canine reproductive cells have not been tested yet, some attempts were made in other species. For instance, IVM of pig oocytes in a polydimethylsiloxane (PDMS) microchannel device gave the same results as the 2D control in terms of MII rates [149], but it improved embryo cleavage [150]. Similarly, IVM of bovine oocytes was also feasible in a static microfluidic device [151]. Besides, IVC of human [150] and mouse [151] embryos in microfluidic devices improved blastocyst development. The use of microfluidic systems enriched with companion cells was also tested, and it improved blastocyst rate in mice [152], and the use of a combination of 3D hydrogels and microfluidics would also be possible [153]. While some chips are designed to be used in a lab setting, others could be employable in the field and might also be useful for assisted reproduction in wildlife species [154]. Current efforts are directed towards the creation of “All-in-one” systems, where the whole IVEP could be performed without unnecessary oocyte and embryo manipulation [155,156].

It is also worth highlighting that, in cats and dogs, innovative systems (3D and microfluidic) were already used for follicle culture [157,158,159,160], but this topic lies outside the scope of this review.

Speaking altogether of the innovative culture systems discussed so far, some other considerations should be made. Although these culture systems have less issues with experimental variability, compared to co-cultures, they are still not completely standardized [71], since there could be some differences in the scaffolds, which are mostly lab-made. In addition, biomaterials themselves can lead to some uncertainty because of their less defined molecular composition and of batch differences [72]. Thus, the design of more easily reproducible 3D culture systems for oocytes and embryos is a matter of great interest. A recent example of more reproducible 3D culture systems used for oocyte culture in other species is based on the use of 3D printing. This is an innovative tissue engineering method that has a huge potential to create scaffolds with controlled shape, size, geometry, porosity and other physical and biochemical features [161]. Bioprinted alginate-based microbeads were created with a spherical hydrogel generator and used for the IVM of sheep oocytes, resulting in an increase in maturation rates, an improvement of oocyte bioenergetic/oxidative status and a modulation of gene expression [162]. This method is strongly reproducible and allows COCs integrity in a more physiological environment: further studies on 3D IVEP should be heading in this direction.

## 5. Conclusions

Considering the challenges in cat and, especially, dog IVEP, alternative and innovative culture systems could offer a chance to obtain better outcomes. State of the art 3D and microfluidic culture systems could resemble more accurately the in vivo growth conditions and help to maintain oocyte and embryo natural morpho-physiology. Although some positive results have been obtained, more investigations on the actual effects of these systems on cat and dog reproductive cells are warranted in order to design improved, ad hoc, enriched culture conditions for domestic carnivore IVEP.

## Data Availability

No new data were created or analyzed in this study. Data sharing is not applicable to this article.
